# Sodium-glucose co-transporter (SGLT) inhibitor restores lost axonal varicosities of the myenteric plexus in a mouse model of high-fat diet-induced obesity

**DOI:** 10.1038/s41598-020-69256-9

**Published:** 2020-07-23

**Authors:** Satoshi Shimo, Sei Saitoh, Huy Bang Nguyen, Truc Quynh Thai, Masako Ikutomo, Ken Muramatsu, Nobuhiko Ohno

**Affiliations:** 1 Department of Occupational Therapy, Health Science University, 7187 Kodachi, Fujikawaguchiko, Yamanashi 401-0380 Japan; 2 Department of Anatomy II and Cell Biology, Fujita Health University School of Medicine, 1-98 Dengakugakubo, Kutsukake-cho, Toyoake, Aichi 470-1192 Japan; 3 Division of Neurobiology and Bioinformatics, National Institute for Physiological Sciences, 38 Saigonaka, Meidaiji-cho, Okazaki, Aichi 444-8787 Japan; 4 Department of Anatomy and Structural Biology, Graduate School of Medical Science, University of Yamanashi, 1110 Shimokato, Chuo, Yamanashi 409-3898 Japan; 5 Department of Anatomy, Faculty of Medicine, University of Medicine and Pharmacy (UMP), 217 Hong Bang, District 5, Ho Chi Minh, 70000 Vietnam; 6 Department of Histology Embryology Genetics, Faculty of Basic Medical Sciences, Pham Ngoc Thach University of Medicine, 02 Duong Quang Trung, District 10, Ho Chi Minh, 70000 Vietnam; 7 Department of Physical Therapy, University of Tokyo Health Sciences, 4-11 Ochiai, Tama, Tokyo 206-0033 Japan; 8 Department of Physical Therapy, Kyorin University Faculty of Health Sciences, 5-4-1 Shimorenjaku, Mitaka, Tokyo 181-8612 Japan; 9 Department of Anatomy, Division of Histology and Cell Biology, School of Medicine, Jichi Medical University, 3311-1 Yakushiji, Shimotsuke, Tochigi 329-0498 Japan

**Keywords:** Enteric nervous system, Enteric neuropathies

## Abstract

Diabetes impairs enteric nervous system functions; however, ultrastructural changes underlying the pathophysiology of the myenteric plexus and the effects of sodium-glucose co-transporter (SGLT) inhibitors are poorly understood. This study aimed to investigate three-dimensional ultrastructural changes in axonal varicosities in the myenteric plexus and the effect thereon of the SGLT inhibitor phlorizin in mice fed a high-fat diet (HFD). Three-dimensional ultrastructural analysis using serial block-face imaging revealed that non-treated HFD-fed mice had fewer axonal varicosities and synaptic vesicles in the myenteric plexus than did normal diet-fed control mice. Furthermore, mitochondrial volume was increased and lysosome number decreased in the axons of non-treated HFD-fed mice when compared to those of control mice. Phlorizin treatment restored the axonal varicosities and organelles in HFD-fed mice. Although HFD did not affect the immunolocalisation of PGP9.5, it reduced synaptophysin immunostaining in the myenteric plexus, which was restored by phlorizin treatment. These results suggest that impairment of the axonal varicosities and their synaptic vesicles underlies the damage to the enteric neurons caused by HFD feeding. SGLT inhibitor treatment could restore axonal varicosities and organelles, which may lead to improved gastrointestinal functions in HFD-induced obesity as well as diabetes.

## Introduction

The enteric nervous system (ENS) is mainly composed of the myenteric and the submucosal plexus, the former of which is located between the longitudinal and circular smooth muscle layers, and regulates the physiological functions of the gastrointestinal tract^1^. Altered ENS function is considered to be involved in the pathogenesis of several digestive system disorders. For instance, gastrointestinal motility disorders, such as vomiting, constipation, diarrhoea, and faecal incontinence, often accompany diabetes. Up to 75% of patients with diabetes experience symptoms of autonomic neuropathies^2,3^, in which hyperglycaemia increases glucose metabolism via the polyol pathway by enhancing inflammation-induced oxidative stress and dyslipidaemia^4^. Importantly, a change of this nature in the gastrointestinal nutrient flow is likely to exacerbate the existing dysfunction of whole-body metabolism and glucose regulation in patients^5^.

High-fat diet (HFD)-ingesting animals have been used to simulate western diet-induced prediabetes and type 2 diabetes mellitus (T2DM) in humans^6,7^. In rodents fed an HFD, peripheral neuropathy, reflected by a decrease in motor and sensory nerve conduction velocity and impairment in behavioural responses to mechanical and thermal stimuli, is observed^8,9^. Further, an HFD has been found to increase ROS production and reduce antioxidant enzyme activities, with a concurrent accumulation of oxidatively damaged mitochondrial proteins and increased mitochondrial fission^10^. These results suggest that mitochondrial damage and dysfunction may play a role in the dying-back neurodegeneration that occurs in diabetic neuropathy. Although the above studies suggested that increased dietary fat predisposes animals to nerve dysfunction even in the absence of T2DM. However, whether such neurological changes occur in the autonomic nerves of the intestinal tract in response to an HFD remains unknown, and the longstanding structural changes in the myenteric plexus caused by HFD have not been fully characterised via detailed ultrastructural analyses^11^.

Different therapeutic and/or preventive strategies for enteric neuropathy, including the use of insulin, nerve growth factor or antioxidants, as well as myenteric neuron transplantation, have been proposed^12^. While several treatments for enteric neuropathy are available, more effective treatment strategies are needed. Furthermore, a greater understanding of myenteric plexus morphological and ultrastructural changes associated with enteric neuropathy may facilitate the development of new strategies. Recently, the use of sodium-glucose co-transporter (SGLT) inhibitors to lower blood glucose levels in patients with diabetes by inhibiting sugar reabsorption has been suggested as a treatment approach. The principal pharmacological action of SGLT inhibitors is the induction of renal glycosuria and blockade of intestinal glucose absorption via inhibition of sodium-glucose transporters in the proximal renal tubule and in the mucosa of the small intestine^13,14^. Currently, little is known about the effect of SGLT inhibitors on the myenteric plexus in HFD-induced obesity as well as diabetes.

The structure of the myenteric plexus is complex, which makes it difficult to elucidate its three-dimensional (3D) morphology using conventional electron microscopes. We used serial block-face scanning electron microscopy (SBF-SEM), which enables much more efficient acquisition of a series of ultrastructural images^15^, to analyse the ultrastructural changes in neurons and the processes of the myenteric plexus in a mouse model of HFD-induced obesity. In addition, we evaluated the effect thereon of an SGLT inhibitor, phlorizin (PLZ), via immunohistochemistry and 3D ultrastructural analyses.

## Results

### Obesity and PLZ-responsive hyperglycaemia in 60% HFD-fed mice

**Figure 1:**
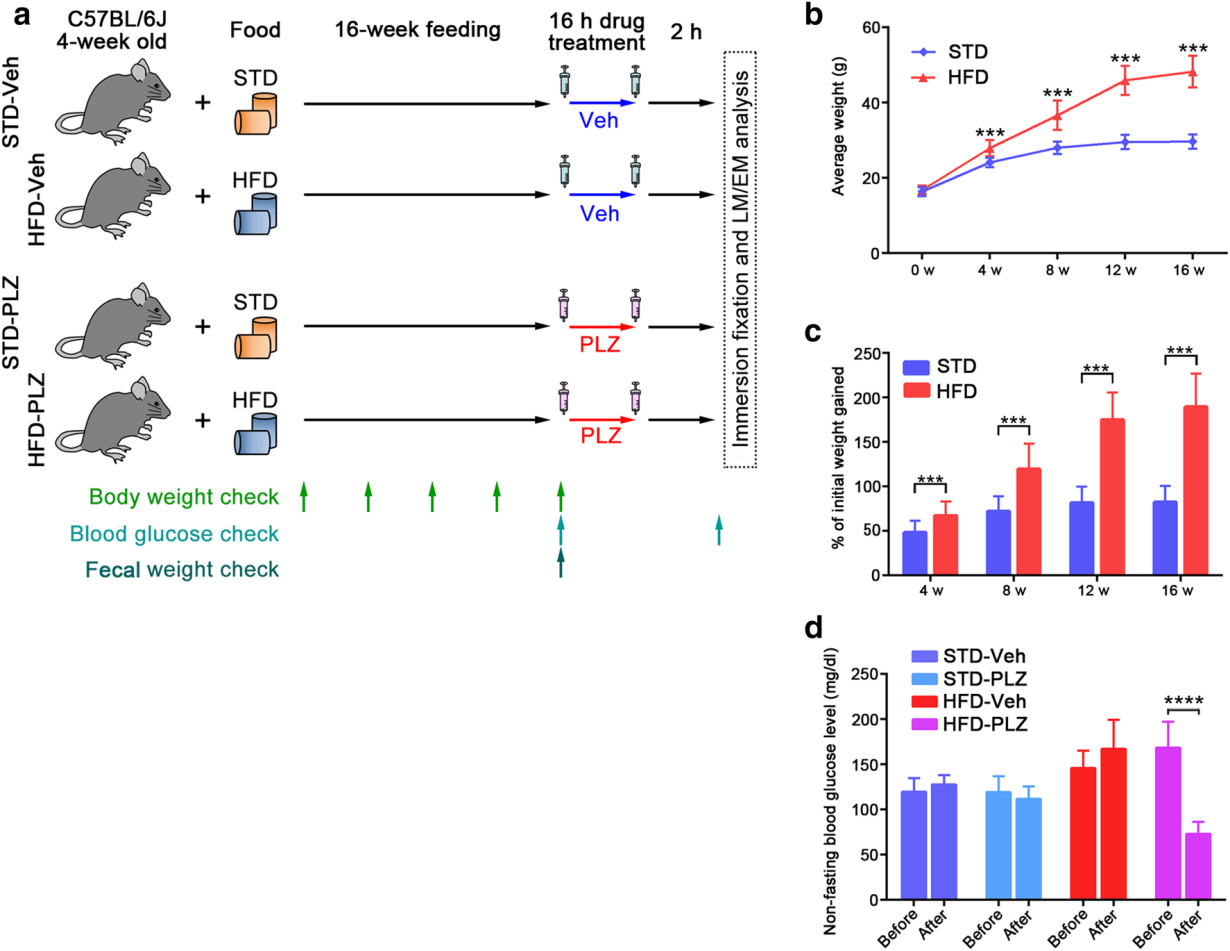
Phlorizin (PLZ) ameliorates increased blood glucose levels in high-fat diet (HFD)-fed mice. (**a**) Experimental design. (**b**) Weight gain in HFD- (n = 16) and standard diet (STD)-fed mice (n = 16) over a 16-week period. (**c**) Weight gain was independent of mouse initial body weight. (**d**) Non-fasting blood glucose levels pre and post vehicle (Veh) (STD-Veh; n = 8, HFD-Veh; n = 8) as well as pre and post PLZ administration (STD-PLZ; n = 8, HFD-PLZ; n = 8). Results are presented as the mean ± SD. ***P < 0.001, ****P < 0.0001 (unpaired Student’s *t-*tests).

When fed an HFD, after 16 weeks, mice showed hallmark symptoms of prediabetes, including increased body weight, impaired glucose tolerance, and abnormal levels of fasting glucose, when compared to mice fed a standard diet (STD)^16,17^. To monitor the progression of obesity induced by HFD feeding, the mice in the HFD- and STD-fed groups were weighed every 4 weeks (Fig. 1a). As the healthy adult mice continued to grow throughout the study period, a gradual increase in weight was maintained by STD-fed mice (Fig. 1b). The weight increase in HFD-fed mice was greater than that in STD-fed mice, and the weights in the two groups differed significantly after 4 weeks (Fig. 1b, *P* < 0.001). HFD-fed mice gained a higher proportion of their initial weights by week 4, and the difference persisted up to week 16 (Fig. 1c; 4–16 weeks, *P* < 0.001). After 16 weeks of PLZ treatment significantly suppressed glucose levels in HFD-fed mice (73 ± 13 mg/dl, n = 8) (*P* < 0.0001), but not in STD-fed mice (Fig. 1d). Blood glucose levels were not altered by vehicle treatment in STD- (STD-Veh) or HFD- (HFD-Veh) fed mice (Fig. 1d). These results demonstrated that the onset of obesity/overweight occurred at 4–16 weeks of HFD intake and was accompanied by an increase in the blood glucose level, which was ameliorated by PLZ treatment.

### 3D reconstruction of synaptic distribution in the axons of the myenteric plexus under HFD with or without PLZ administration

**Figure 2:**
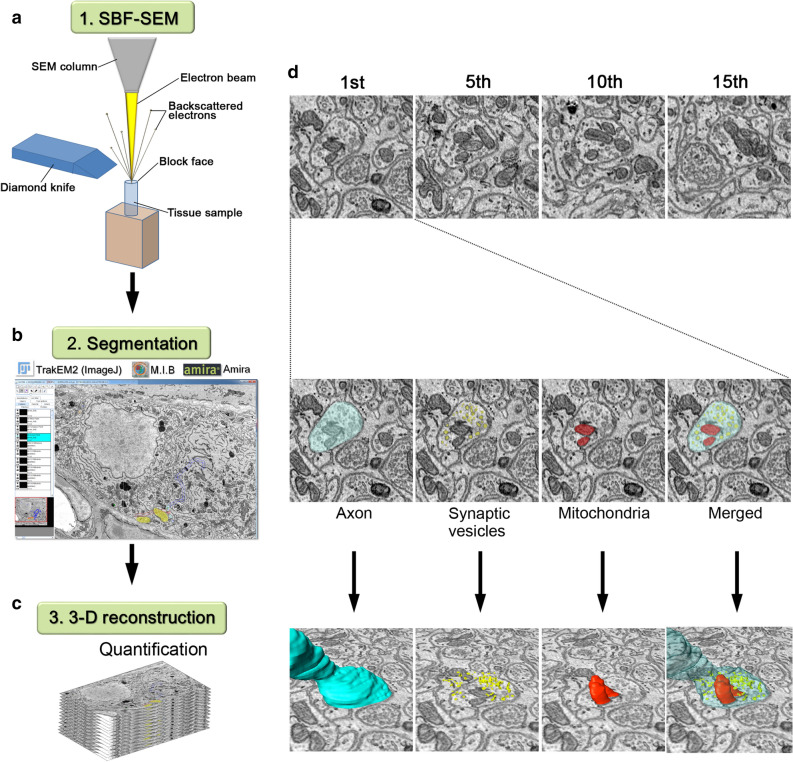
Workflow for 3D reconstruction of axonal varicosities in the myenteric plexus. (**a**) A diamond knife (blue) was used cut 50- or 70-nm ultrathin slices from the top of the block-face in SEM (SBF-SEM). In this method, an image is captured after every retraction of the knife, and the cutting and imaging cycle is automatically repeated to acquire serial images. (**b**) SBF-SEM can produce a series of hundreds or even thousands of orthoslices where segmentation of various ultrastructures can be performed with different software. (**c**) Following acquisitions and semi-automatic or manual segmentation, orthoslice series and structures were rendered into 3D ultrastructure and quantified. (**d**) Top: representative (1st, 5th, 10th and 15th) images from an actual stack at 50-nm thickness. Middle: axon (light blue), synaptic vesicles (yellow), and mitochondria (red) were marked in semi-automatic and manual segmentation, and traced using TrakEM2 (https://imagej.net/TrakEM2), Amira version 5.6 (https://www.fei.com/software/amira/), and Microscopy Image Browser. Bottom: three-dimensionally (3D) rendered axon (blue), synaptic vesicles (yellow), mitochondria (red), and merged image.

**Figure 3:**
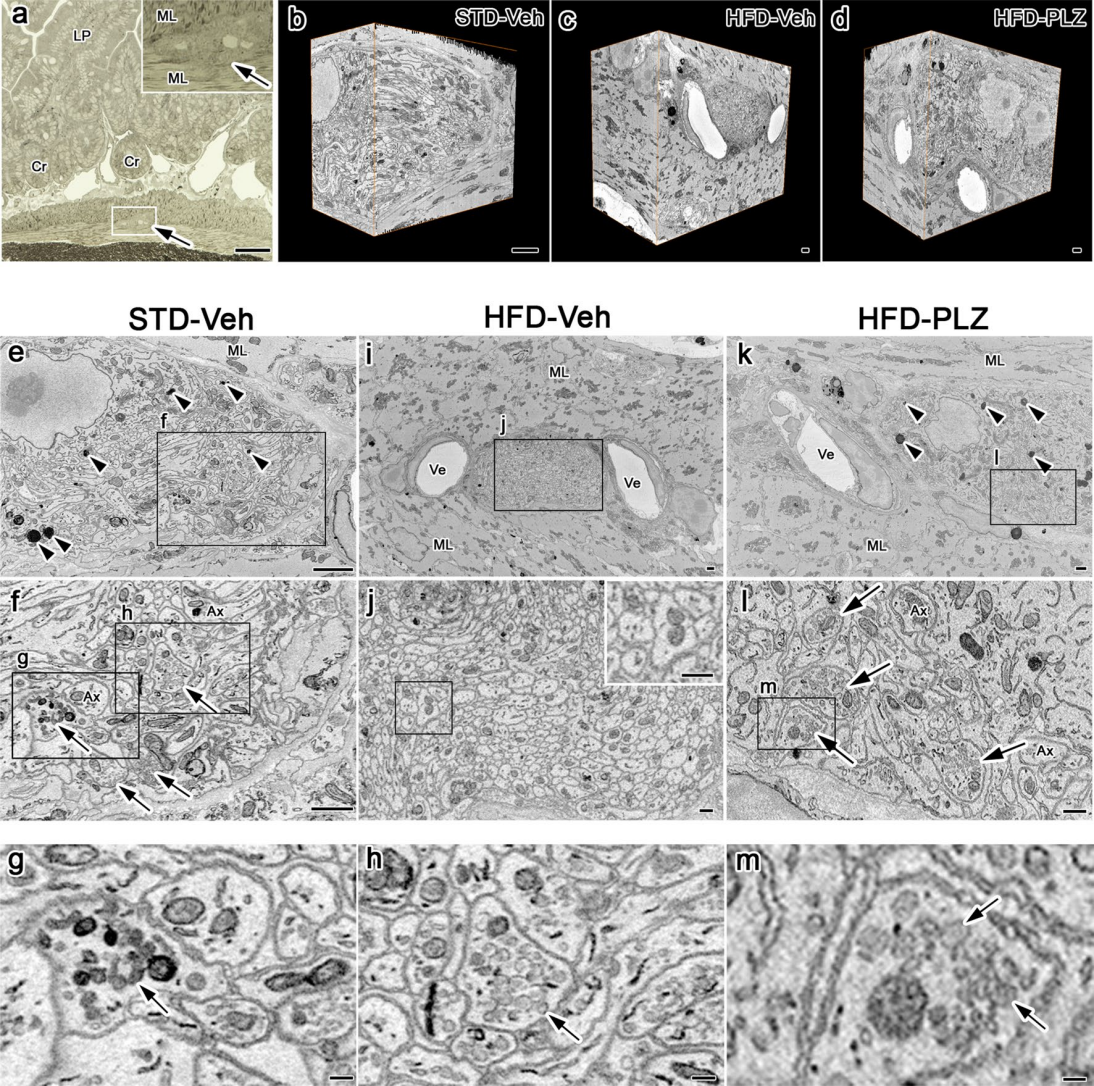
Phlorizin treatment restores loss of axonal varicosities and synaptic vesicles induced by high-fat diet (HFD). (**a**) Light micrograph of 1 µm thick section in STD-fed, vehicle (Veh)-treated mice; arrow, (inset) magnified muscular layer (ML) with myenteric plexus (arrow). (**b**–**d**) 3D reconstructions of serial electron microscopic images of the myenteric plexus in (**b**) STD-Veh, (**c**) HFD-Veh, and (**d**) HFD-PLZ mice. Several lysosomes were located in the myenteric plexus in STD-Veh (**e**, arrowheads) and HFD-PLZ (**k**, arrowheads) mice. (**e**–**h**) STD-Veh mice with varicosities of axons (Ax) with abundant synaptic vesicles (**f**–**h**, arrows). (**i**) Electron micrographs of HFD-Veh mice. (**j**) The myenteric plexus is highly magnified, corresponding to the rectangular areas in (**i**). Inset in the figures show a highly magnified view of the square areas. In the myenteric plexus of HFD-Veh mice, synaptic vesicles in axons were observed less frequently. (**k**–**m**) Axonal varicosities with numerous synaptic vesicles (**l**,**m**, arrows) in HFD-PLZ mice. Cr: crypt, LP: Lamina propria. Bars: (**a**) 50 µm, (**b**–**e**,**i**,**k**) 1 µm, (**f**,**j**,**l**, inset in **j**) 500 nm, (**g**,**h**,**m**) 100 nm.

**Figure 4:**
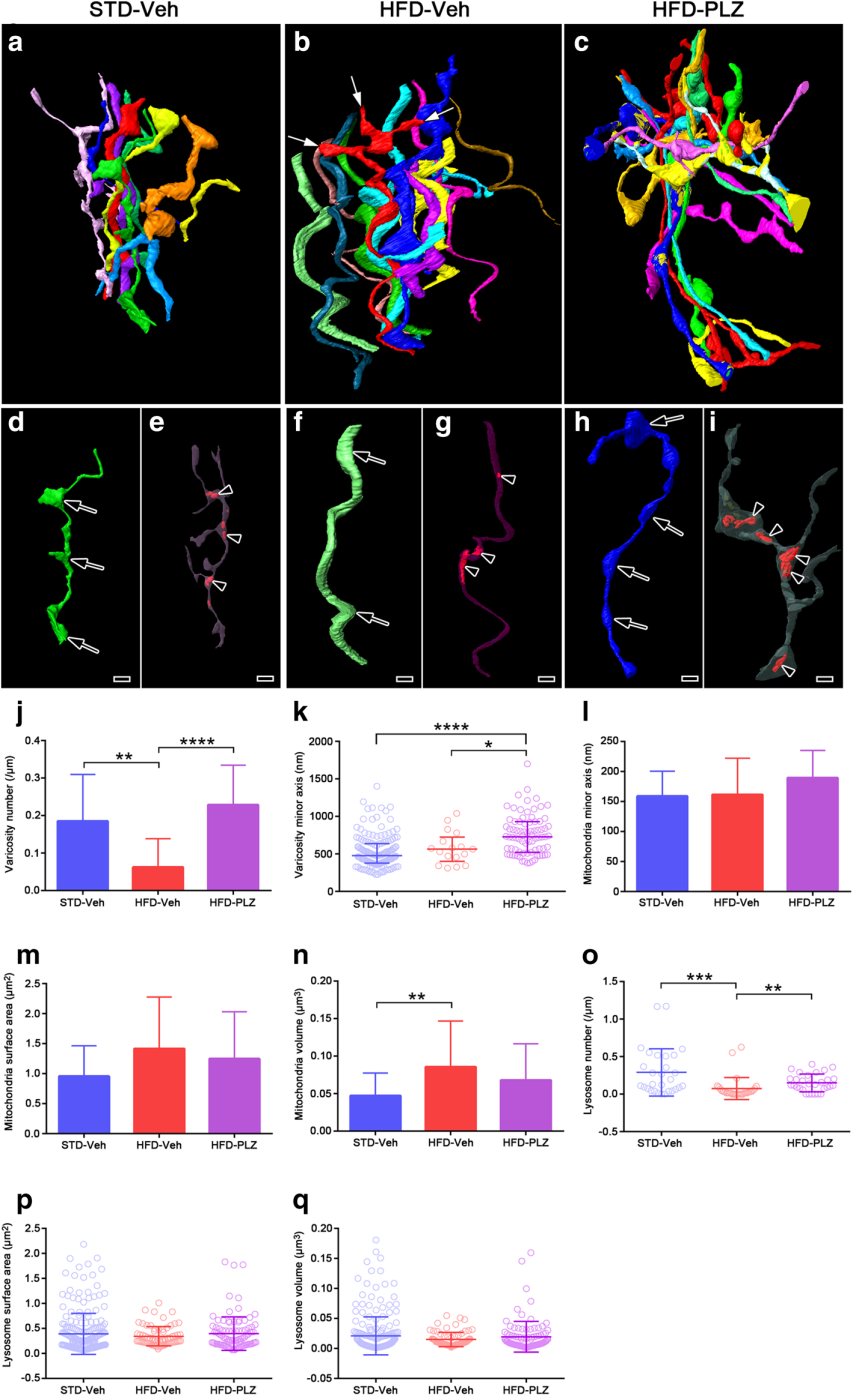
PLZ treatment restores the loss of axonal varicosities under a HFD. Reconstructed 3D volume of axons with varicosities and collateral branches in the myenteric plexus in STD-Veh (**a**,**d**,**e**), HFD-Veh (**b**,**f**,**g**), and HFD-PLZ (**c**,**h**,**i**) mice. Varicosity areas (**d**,**f**,**h**, arrows) and collateral branches (**b**, white arrows) and axonal mitochondria (**e**,**g**,**i**, arrowheads) are shown. (**j**) HFD-Veh mice had fewer axonal varicosities than STD-Veh mice, whereas HFD-PLZ mice contained more axonal varicosities with numerous synaptic vesicles than HFD-Veh mice. (**k**) The varicosity minor axis was increased in HFD-PLZ mice compared to that in STD-Veh and HFD-Veh mice. (**l**) Mitochondrial minor axis and surface area (m) were not significantly different. (**n**) Mitochondrial volume was increased in HFD-Veh mice when compared to that in STD-Veh mice. (**o**) Lysosome number decreased in HFD-PLZ mice compared to that in HFD-Veh mice. n (STD = 3, HFD = 3, HFD-PLZ = 3) = 30 axons each (**j**,**o–q**), 158, 17, 79 varicosities (**k**), or 30 mitochondria (**l**–**n**). Graphs show the mean ± SD (**j**,**l**–**n**) for normally distributed data or the medians (**k**,**o**–**q**, bars) with quartile range (**k**,**o**–**q**, whiskers) for other data. Statistical significances were calculated by ANOVA followed by Bonferroni post-hoc tests (**j**,**l**–**n**) or Kruskal–Wallis tests (**k**,**o**). *P < 0.05, **P < 0.01, ****P < 0.0001. Bars: 1 µm.

To clearly elucidate the ultrastructural changes in the myenteric plexus induced by an HFD, serial electron microscopic images were obtained by SBF-SEM (Fig. 2a). Subsequently, segmentation (Fig. 2b), followed by 3D reconstruction of axons with the organelles was performed for structural quantification (Fig. 2c). In the serial images (Fig. 2d), axons with varicosities were readily identified and were found to contain numerous synaptic vesicles and organelles. The distribution and morphology of these organelles within and outside the varicosities were reconstructed and analysed (Fig. 2d). In the small intestinal muscle layers (Fig. 3a), the myenteric plexus was identified, and serial images of STD-Veh (Fig. 3b), HFD-Veh (Fig. 3c) and HFD-PLZ (Fig. 3d) mouse tissues were acquired 18 h following vehicle or PLZ treatment (Fig. 1a). In the myenteric plexus of STD-Veh mice, the ganglia appeared as highly compact structures occupied by nervous and glial elements (Fig. 3e). The ganglia were completely surrounded by a basal lamina and isolated from the connective tissues and blood vessels (Supplementary Figure S1). Axons were abundant in the varicosities, with different types of synaptic vesicles (arrows in Fig. 3f–h). Furthermore, several mitochondria were located near the synaptic vesicles within the varicosity of STD-Veh mice (Fig. 3g,h). The axons within the ganglia around granular vesicles were presumably the processes of an intramural neuron (Supplementary Figure S2). In the myenteric plexus of HFD-Veh mice, synaptic vesicles in axons were observed less frequently; however, the myenteric axons were rich in mitochondria (Fig. 3i,j). In contrast, we detected numerous synaptic vesicles in the varicosities of the axons in HFD-PLZ mice (Fig. 3k–m). Next, we 3D-reconstructed axons including varicosities and organelles from the serial electron microscopic images of STD-Veh (Fig. 4a), HFD-Veh (Fig. 4b) and HFD-PLZ (Fig. 4c) mice. In these images, we detected differently shaped varicosities (Fig. 4d, arrows), each of which contained numerous synaptic vesicles. Varicosities were commonly observed in axons with different types of vesicles, as also observed in the 2D electron microscopic images (Fig. 3g,h). Some of the mitochondria in these axons were located within varicosities, whereas others were located in the axonal segments among the varicosities (Fig. 4e, arrowheads). Furthermore, in the 3D images, the axons in HFD-Veh mice appeared to have fewer varicosities (Fig. 4f,j) and collateral branches on the axons (Fig. 4b, white arrows). In addition, the mitochondria in HFD-Veh axons appeared to be larger than those in STD-Veh axons (Fig. 4g,n). In contrast, we clearly detected various forms of varicosities in the 3D-reconstructed images of HFD-PLZ mice (Fig. 4h, arrows, j). In addition, mitochondria in HFD-PLZ axons appeared to be smaller than those in HFD-Veh axons (Fig. 4i, arrowheads, m,n). Interestingly, HFD-PLZ mice had more detectable synaptic vesicles in the axonal varicosities, and the varicosity size, measured as the minor axis length, was larger than that in STD-Veh (Fig. 4k, *P* < 0.0001) and HFD-Veh (Fig. 4k, *P* < 0.05) mice. Furthermore, mitochondrial volume was increased in HFD-Veh compared to STD-Veh mice (Fig. 4n, *P* < 0.01). Concomitantly, the lysosome number in HFD-Veh axons was lower than that in STD-Veh and HFD-PLZ axons (Fig. 4o). These results suggested that impairment of axonal varicosities and their synaptic vesicles as well as mitochondrial structure and lysosome number changes may underlie the ENS damages induced by an HFD, and that treatment with SGLT inhibitors could restore the axonal varicosities.

### Immunolocalisation of protein gene product 9.5 (PGP9.5), synaptophysin, and c-Kit in the myenteric plexus under HFD feeding and PLZ treatment

**Figure 5:**
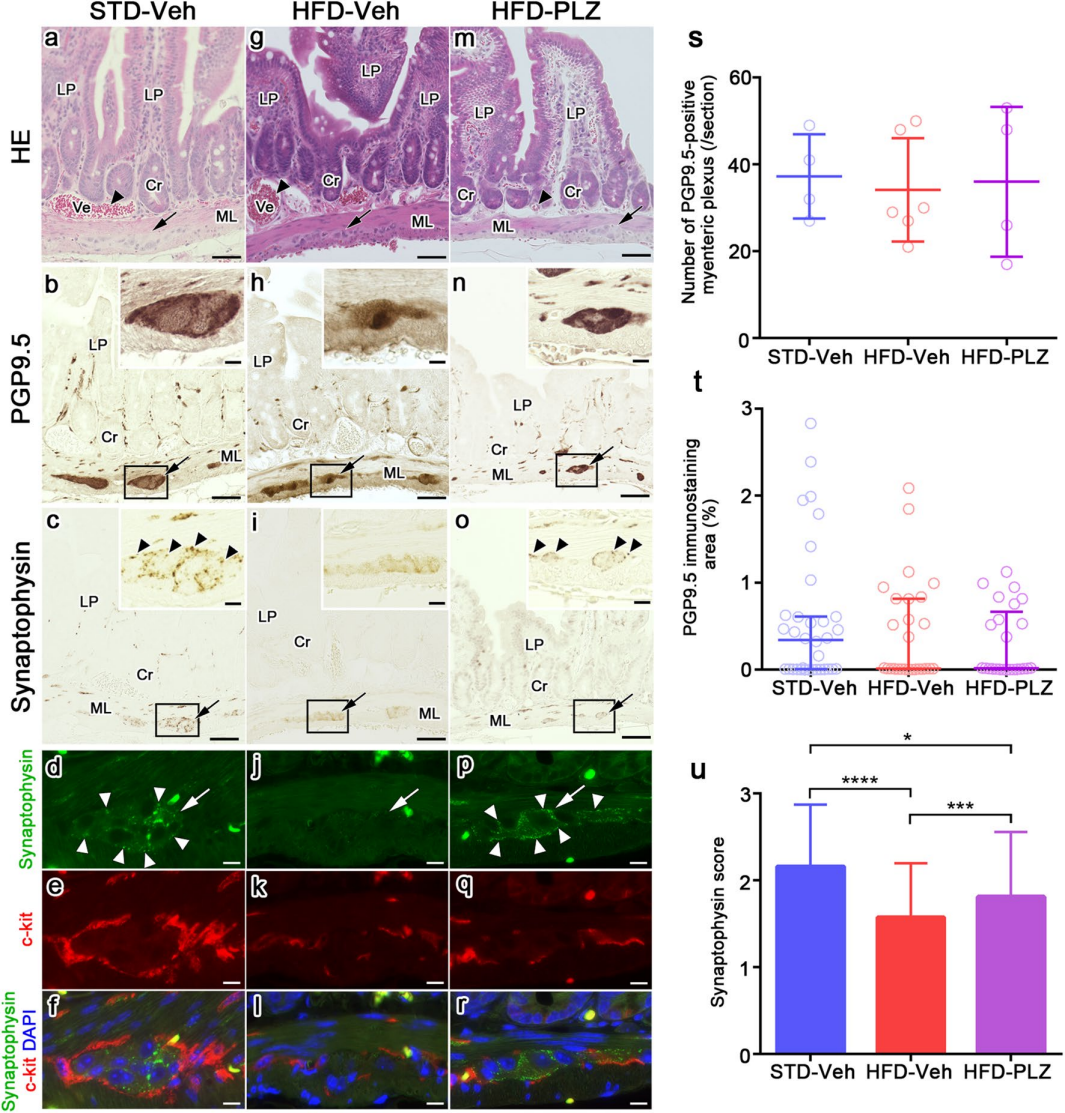
A neuronal marker was unaffected, whereas a presynaptic marker was decreased under HFD, and the presynaptic marker was restored with PLZ treatment. Light micrographs of HE staining (**a**,**g**,**m**) and the neighbouring sections that were immunohistochemically stained for PGP9.5 (**b**,**h**,**n**) and synaptophysin (**c**,**i**,**o**), or double-immunostained for synaptophysin (**d**,**j**,**p**, green) and c-Kit (**e**,**k**,**q**, red) with nuclear labelling (**f**,**l**,**r**, blue, DAPI) in STD-Veh and HFD-Veh mice and HFD-PLZ mice. The myenteric plexus has been indicated by black arrows (**a**-**c**,**g**-**i**,**m**-**o**) and white arrows (**d**,**j**,**p**). Background signal of red blood cells appears yellow (**f**,**l**,**r**). (**s**) The number of PGP9.5-positive ganglionic area and (**t**) PGP9.5-positive % area in individual sections of the myenteric plexus did not differ among the STD-Veh (n = 5), HFD-Veh (n = 5), and HFD-PLZ mice (n = 5). (**u**) The semiquantitative synaptophysin score in HFD-Veh mice was lower than that in STD-Veh mice, but was significantly increased in HFD-PLZ mice. N (STD, HFD, HFD-PLZ) = 4, 6, 4 sections (**s**) or 156, 148, 91 plexi (**u**). Results are presented as the mean ± SD for normally distributed data or the median (**s**,**t**, bars) with quartile range (**s**,**t**, whiskers) for other data. Statistical significances were calculated by ANOVA followed by Kruskal–Wallis tests (**s**,**t**) or Bonferroni post-hoc tests (**u**). *P < 0.05, ***P < 0.001, ****P < 0.0001. Cr: crypt, LP: Lamina propria, ML: muscular layer, Ve: blood vessel (**a**,**g**,**m**, arrowheads). Bars: 50 µm (**a**–**c**,**g**–**i**,**m**–**o**), 10 µm (others).

To examine the observed changes in axonal varicosities further, we analysed the immunolocalisation of PGP9.5, synaptophysin, and c-Kit in adjacent paraffin sections. In small intestinal tissues of STD-Veh mice (Fig. 5a), PGP9.5 immunoreactivity was strongly detected in the myenteric plexus (Fig. 5b, arrow). In addition, small bundles of PGP9.5 formed a continuous network in the connective tissues of the lamina propria (Fig. 5b). Synaptophysin immunoreactivity was clearly observed around the myenteric plexus in the muscular layers of STD-Veh mice (Fig. 5c, arrowheads). Double immunostaining for synaptophysin and c-kit revealed that synaptophysin immunoreactivity (Fig. 5d, white arrows) localised in the myenteric plexus was surrounded by c-kit-positive interstitial cells of Cajal (Fig. 5e–f). In the small intestines of HFD-Veh mice (Fig. 5g), the immunolocalisation of PGP9.5 was similar to that in the STD-Veh mice (Fig. 5h, arrow). However, the immunoreactivity of synaptophysin was significantly decreased (Fig. 5i, inset), and was barely detectable in the myenteric plexus (Fig. 5j, white arrow) surrounded by c-kit immunoreactivity (Fig. 5k–l). Conversely, in HFD-PLZ mice (Fig. 5m), while the immunolocalisation of PGP9.5 was similar to that in the HFD-Veh group, synaptophysin immunoreactivity was clearly detected (Fig. 5o, arrowheads and Fig. 5p, white arrowheads) in the myenteric plexus. In quantitative analyses, the number and percentage area of PGP9.5-positive myenteric plexi per transverse section did not significantly differ among the STD-Veh, HFD-Veh, and HFD-PLZ groups (Fig. 5s,t). However, semiquantitative analysis of the synaptophysin immunoreactivity revealed that it was significantly decreased in the HFD-Veh compared to the STD-Veh group (Fig. 5u, *P* < 0.0001). In addition, synaptophysin immunoreactivity was higher in the HFD-PLZ than in the HFD-Veh group, although it remained lower than that in the STD-Veh group (Fig. 5u, *P* < 0.001).

**Figure 6:**
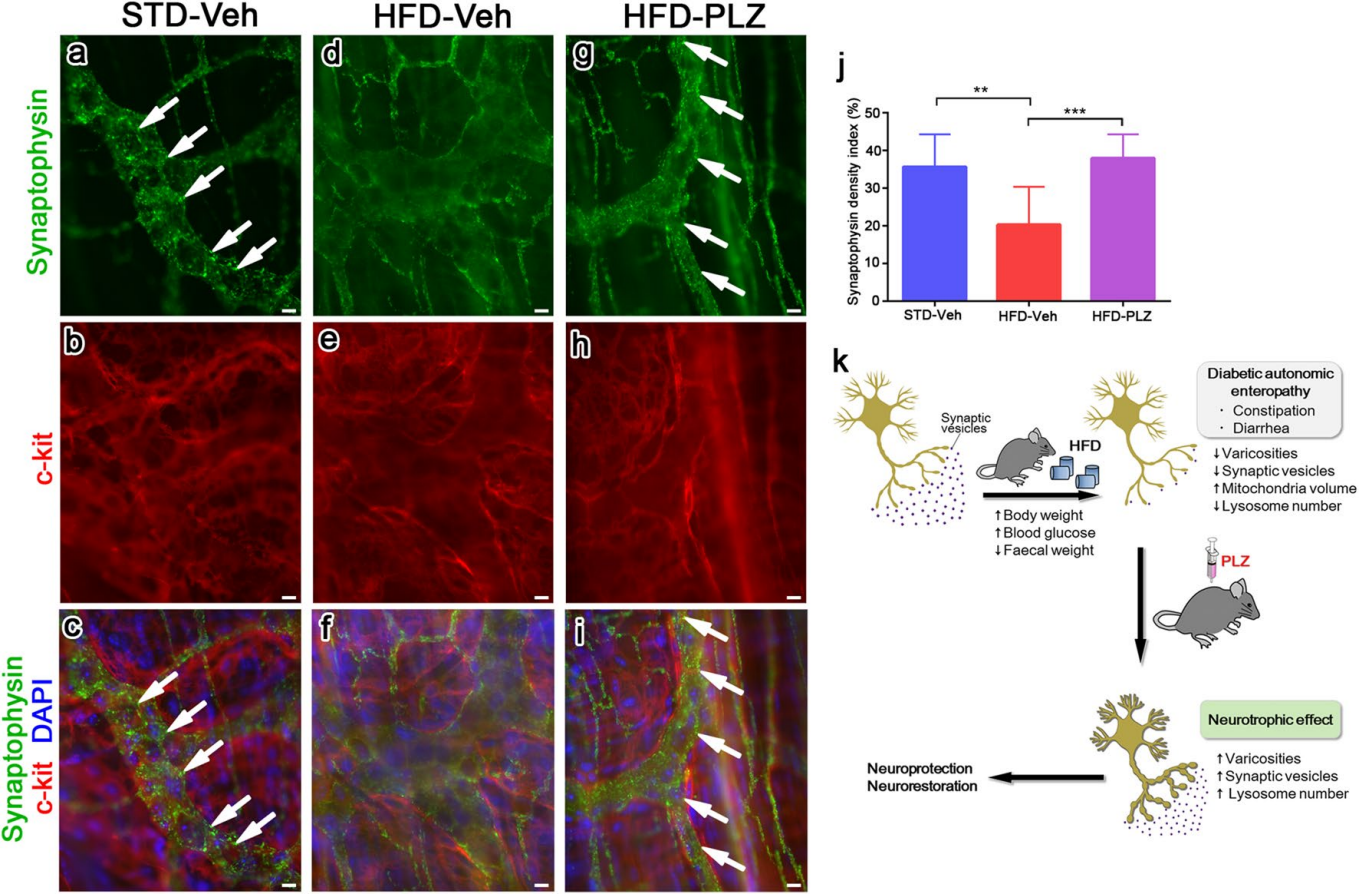
Myenteric plexus of the mouse small intestine stained by the whole-mount preparation method. Double immunofluorescence staining for synaptophysin (green) and c-Kit (red) in STD-Veh (**a**–**c**), HFD-Veh (**d**–**f**), and HFD-PLZ mice (**g**–**i**). Nuclei were counterstained with DAPI (**c**,**f**,**i**, blue). Synaptophysin immunoreactivities in STD-Veh (**a**,**c**, white arrows) and HFD-PLZ mice (**g**,**i**, white arrows) were clearly observed as numerous small granular immunoreaction signals in the myenteric plexus. (**j**) In HFD-Veh mice (n = 3), the synaptophysin density index (stained area/total myenteric area) was lower than that in STD-Veh mice (n = 3), but it was significantly increased in HFD-PLZ mice (n = 3). (**k**) Graphical summary of the model. Results are presented as the mean ± SD. Statistical significances were calculated by ANOVA followed by Bonferroni post-hoc tests. **P < 0.01, ***P < 0.001. Bars: 10 µm.

To correlate the 3D ultrastructural reconstruction of large tissue areas and light microscopic observation of immunofluorescence in single paraffin sections, we used immunohistochemical analyses of the whole-mount preparation. The results showed that the distribution of neuronal projections could be visualised and the immunodistribution of specific molecules could be examined in the network of nerve fibres in the myenteric plexus. In these analyses, synaptophysin immunoreactivities in STD-Veh mice were clearly observed as numerous small granular immunoreaction signals in the myenteric plexus where myenteric neurons were linearly aligned along the granular immunoreactivity (Fig. 6a,c, white arrows). In HFD-Veh mice, synaptophysin immunoreaction signals were thin and significantly reduced in the myenteric plexus demarcated by c-kit-immunopositive interstitial cells of Cajal (Fig. 6d,f). In contrast, synaptophysin immunoreactivities in HFD-PLZ mice were dense, and their small granular immunoreaction signals were more clearly observed in the myenteric plexus (Fig. 6g,i, white arrows). These changes were supported by quantitative analyses of the synaptophysin density index (stained area/myenteric area), and the index of HFD-PLZ was significantly increased compared with that of HFD-Veh (Fig. 6j). Collectively, these results demonstrated that, although PGP9.5-positive myenteric neurons and their axons are well maintained under HFD feeding, synaptophysin-positive synaptic vesicles are drastically decreased, and short-term PLZ treatment can substantially restore synaptophysin-positive synaptic varicosities in neurons of the myenteric plexus in HFD-fed mice.

## Discussion

In the present study, we used a mouse model of prediabetes established by long-term consumption of an HFD to analyse changes in neuronal processes and the effect of an SGLT inhibitor on neurons in the myenteric plexus. 3D ultrastructural analyses of the myenteric plexus revealed that the axons in HFD-Veh mice had fewer varicosities and collateral branches when compared with those in STD-Veh mice. In addition, mitochondria of HFD-Veh were larger than those of STD-Veh. Interestingly, the varicosity size, was larger in the HFD-PLZ group than in STD-Veh and HFD-Veh groups. Although numbers of PGP9.5-immunopositive neurons and their processes in the myenteric plexus did not significantly differ among the STD-Veh, HFD-Veh, and HFD-PLZ groups, synaptophysin immunoreactivity in the myenteric plexus was significantly lower in the HFD-Veh group than in the STD-Veh and HFD-PLZ groups. These results suggest that abnormalities in the axonal varicosities and their synaptic vesicles, along with mitochondrial remodelling, underlie the HFD-induced ENS damages. Furthermore, the results support the proposal that treatment with SGLT inhibitors can restore axonal varicosities and may lead to improved gastrointestinal functions in HFD-induced obesity and diabetes.

Prediabetes is currently recognised as a key contributory factor associated with the development of idiopathic neuropathy in nondiabetic patients^18–20^; however, the pathogenic mechanisms underlying diabetic peripheral neuropathy development in the context of prediabetes remain undefined. Unlike db/db and ob/ob mice, which are diabetic from 4 weeks of age and essentially bypass prediabetes, HFD-fed mice have a gradual onset of metabolic imbalances that are more characteristic of the human condition, including increased weight gain, impaired glucose tolerance, and decreased motor and sensory nerve conduction velocity^21,22^. As these conditions are representative of prediabetes in humans, this model may be useful to address the causes of the pathology in the myenteric plexus and potential benefits of early therapeutic interventions. However, the dietary composition, including fat content, source, and type, differs among reports, and further investigation is required to determine whether specific HFD contents could predominantly affect the myenteric plexus in HFD-induced prediabetes models.

The HFD-fed mice experienced an increase in nerve injury, including loss of varicosities. Marked axonal degeneration in the myenteric plexus followed by axon sprouting and regeneration has been previously reported in streptozotocin-induced diabetic rats^23,24^. These findings suggest that there are two phases of neuronal changes in diabetes: an initial phase of neuronal loss, and a later phase of regeneration. In contrast, our results showed that, although axonal branches were decreased, neurons and their processes were relatively well maintained in HFD-Veh. These differences may be due to the gradual onset and mild severity of the high blood glucose level under HFD feeding. Conversely, marked changes in specific enteric neuropeptides, such as NPY, nNOS, VIP, CGRP, ChAT, and SP, and a decrease in synaptophysin in the ENS have been observed in experimental models of diabetes^25,26^. In addition, previous studies have reported that degradation of total synaptophysin is accelerated in 2-month-old diabetic rats^27^. Moreover, diabetes is known to increase mRNA translation for select proteins in response to elevated glucose^28^. Increased mRNA translation and accelerated degradation of synaptophysin may be compensatory mechanisms for the observed reduction of the mature protein. We also report for the first time the novel finding that PLZ injection restored synaptic vesicles. These results indicate that phlorizin may reduce synaptophysin degradation and normalize levels of mRNA translation and mature protein, although specific details of the mechanism remain unknown, and further research is required. Our result showing decreased synaptophysin expression in the myenteric plexus in HFD-Veh mice indicates that abnormal production and transport of synaptic neurotransmitters may underlie the neuronal functional impairment in prediabetes. In addition, the presence of large and small varicosities with synaptic vesicles in the HFD-PLZ-fed mice suggests that SGLTs play essential roles in the maintenance of synaptic varicosities and vesicles along the axons. The myenteric plexus includes sensory neurons, interneurons, and excitatory and inhibitory motor neurons that are defined by a specific complement of neural proteins and transmitters. The presence of nNOS, which induces apoptosis in inhibitory neurons, has been proposed to exacerbate oxidative stress caused by the formation of advanced glycation end products^29,30^. Oxidative stress of this nature has also been shown to cause gastrointestinal motor dysfunction^31^. However, addressing the cellular mechanisms underlying enteric nerve damage was not the goal of the present study. Future analyses focusing on the differential effects of HFD feeding and PLZ treatment on the different types of neurons and enteric neuropeptides would be necessary to gain a deeper understanding of the therapeutic potential of SGLT inhibitors in enteropathy under diabetes and prediabetes.

In the present study, PLZ pre-treatment, which reduced the blood glucose levels, reversed the synaptic changes observed in HFD-fed mice. Consistent herewith, mizagliflozin, which potently and selectively inhibits human SGLT1, increased stool frequency and loosened stool consistency under chronic constipation^32^, indicating that SGLT1 inhibition results in enhanced gastrointestinal motility. Interestingly, SGLT3 is found in cholinergic neurons of the submucosal and myenteric plexuses, and glucose transport through SGLT3, which potentially upregulates electrical activity of neurons in a glucose-dependent manner, is blocked by the general SGLT blocker PLZ^33,34^. Therefore, while PLZ reduces blood glucose levels via SGLT1 and SGLT2, PLZ may also directly affect the function of the myenteric plexus^35^. The current study indicates that SGLT inhibitors have therapeutic potential in diabetes by ameliorating synaptic pathology in the myenteric plexus. In the present study, PLZ was administered at 400 mg/kg for each dose, equivalent to approximately 20 g in the case of a human (from a simple calculation assuming 50 kg body weight). Currently, PLZ alone has not been clinically administered in humans, and it is unclear what effect and pharmacokinetics this dose will show. On the other hand, the hypoglycaemic activity of powdered unripe apples (equivalent to approximately 315 mg of phlorizin) or apple extract (approximately 450 mg of phlorizin) in humans has been demonstrated in previous reports^36,37^. However, the majority of study designs have utilized whole fruit or fruit products (e.g. juice), making it difficult to distinguish the effects of PLZ from those of other fruit components. If in the future, the effects of PLZ can be further clarified, it may prove more effective to ingest the PLZ supplement than to ingest it from fruit/fruit products. In time, a comparison with other diabetes drugs, such as metformin, DPP-4 inhibitors, and insulin, as well as a direct examination of the gastrointestinal tract’s physiological functions, including contractility and slow-wave frequency, may provide further information regarding the blood glucose lowering effects of SGLT inhibition and the direct effect of SGLT inhibition on myenteric neurons. In addition, further studies are necessary to clarify the differential roles of SGLT1, SGLT2, and SGLT3 in the changes in the myenteric plexus under pre-diabetic and diabetic conditions.

In addition to the ultrastructural alterations of synaptic varicosities, the current study demonstrated mitochondrial elongation, which suggested increased mitochondrial fusion, in the processes of myenteric neurons under HFD feeding. Mitochondrial morphology and lysosomes are closely associated with cellular metabolism and functions in neurons, and mitochondrial fragmentation has been reported to occur in progressive nerve damage associated with hyperglycaemia^38–41^. The difference between mitochondrial fusion in the myenteric plexus observed in this study and mitochondrial fragmentation in sensory nerves reported previously may suggest variable mitochondrial dynamics responses in different types of neurons under hyperglycaemia. Mitochondrial networks can reduce energy consumption to maintain mitochondrial activity^42^. PGP9.5-positive myenteric neurons were relatively maintained under HFD feeding. Enhanced mitochondrial enlargement and decreased lysosome number in the myenteric neurons of HFD-fed mice could be an adaptive response to hyperglycaemia, supportive of neuronal survival. The concept of mitochondrial enlargement under hyperglycaemia was supported by the findings that PLZ treatment to lower the blood glucose level tended to reduce the mitochondrial size and increase the lysosome number. It is possible that mitochondrial metabolism in the neuronal processes in the myenteric plexus is closely related to the changes in varicosities and synaptic vesicle localisation. However, further studies are necessary to explore the possibility that the mitochondrial structures and dynamics are altered in a time-dependent manner in the different phases of the prediabetes mouse model.

## Methods

### Animals

All animal experiments were performed in accordance with the National Institutes of Health Guide for Care and Use of Laboratory Animals (NIH Publications No. 8023, revised 1978). The animal experiments were approved by the Animal Ethics Committee of Health Science University (approval numbers: #27-25, #28-12, #29-10). Thirty-two C57BL/6 mice (Jackson laboratory No. 000664) were maintained under a 12-h light/dark cycle at 24 °C and fed a standard, low-fat laboratory diet ad libitum. Four-week-old mice were randomly divided into two groups. Each group was placed in a single cage and fed either a STD (n = 16) or an HFD (n = 16) containing 60 kcal% fat, 20 kcal% carbohydrate, and 20 kcal% protein (D12492; Research Diets, New Brunswick, NJ; Table 1)^43,44^. The control mice were sex- and age-matched and were fed standard chow ad libitum. Body weights were monitored every 4 weeks, and after 16 weeks on the assigned diets. Then, the mice were injected subcutaneously with 400 mg/kg of PLZ (FUJIFILM Wako Pure Chemical Corp., Osaka, Japan) [dissolved in 10% EtOH, 15% dimethyl sulfoxide (DMSO), and 75% normal saline (0.9% w/v NaCl)] (STD-PLZ, n = 8; HFD-PLZ, n = 8) or buffer only (STD-Veh, n = 8; HFD-Veh, n = 8) at 16 h and 2 h prior to organ removal. The doses used in this study were selected based on results from previous studies^45,46^. Non-fasting blood glucose concentrations were assessed before and after test injections^47^, via tail puncture, using a blood glucose meter (Abbott, Tokyo, Japan), as shown in Fig. 1a.

**Table 1:** Composition of experimental diets.

Component	Amount (kcal%)	
	STD^a^	HFD^b^
Protein	25.8	20
Carbohydrate	61.9	20
Fat	12.3	60
Total	100	100

^a^ The standard diet (STD) provided was: MF (Oriental Yeast Co., Tokyo, Japan).

^b^ The high-fat diet (HFD) provided was: D12492 (Research Diets, New Brunswick, NJ).

### Tissue preparation

C57BL/6 mice were anaesthetised with an intraperitoneal injection of combined anaesthetics (0.3 mg/kg medetomidine, 4.0 mg/kg midazolam, and 5.0 mg/kg butorphanol). Small intestinal tissues, 2–3 cm from the end of the stomach, were prepared for electron microscopy, and for immunohistochemical analysis using different preparation methods.

### Tissue preparation for SBF-SEM

Some sections of the freshly resected intestinal tissues were cut into small pieces (< 1 mm in size) with a razor and incubated with 4% paraformaldehyde and 1% glutaraldehyde in 0.1 M phosphate buffer (pH 7.4) at 4 °C overnight. En-bloc heavy metal staining was performed as reported previously^48^. Then, the tissues were washed with phosphate-buffered saline (PBS) and used for specimen preparation. The tissues were treated with 2% OsO_4_ in 0.15% K_4_[Fe(CN)_6_] for 1 h on ice, 0.1% thiocarbohydrazide for 20 min, and 2% OsO_4_ for 30 min at room temperature, as previously described^49^. Then, the tissues were incubated in lead aspartate solution at 60 °C for 30 min. Each of the treatments was followed by four rounds of washing with double-distilled water. The tissues were dehydrated using a graded series of ethanol and infiltrated with acetone dehydrated with a molecular sieve of a 1:1 mixture of resin and acetone, followed by 100% resin. Resins were prepared from the Quetol 812 kit (Nisshin EM, Tokyo, Japan) by mixing Quetol 812, DDSA, MNA, and DMP-30, or plain resin following the manufacturer’s instructions, and 7% (w/v) (for Quetol 812) or 17%–19% (w/v) (for Plain resin) carbon black was added to increase the resin conductivity^50^. The samples were embedded in the resins in moulds, and cured at 60 °C overnight.

### SBF-SEM and data analyses

Blocks from each group were trimmed and mounted on aluminium rivets with conductive glue (Chemtronics, Kennesaw, GA, USA). The surfaces of the trimmed samples were sputtered with gold to increase the conductivity and then imaged under various imaging conditions in a MERLIN or SIGMA/VP SEM instrument (Carl Zeiss Microscopy, Jena, Germany) equipped with a 3View in-chamber ultramicrotome (Gatan Inc., Pleasanton, CA, USA). Imaging in the MERLIN instrument was performed under a constant probe current (150 pA) and in the crossover-free mode. Imaging in the Sigma instrument was performed using a 30-μm aperture. The serial images obtained were processed with ImageJ and Fiji plugins (https://fiji.sc/wiki/index.php/Fiji), and segmentation and image analyses were performed in TrakEM2^51^, Amira version 5.6 (FEI Visualisation Science Group, Hillsboro, OR, USA), and Microscopy Image Browser (https://mib.helsinki.fi/), as shown in Fig. 2. Axons, synaptic vesicles, mitochondria, and lysosomes were semi-automatically and manually traced using these software packages.

### Paraffin sections

The small intestines of three anaesthetised mice were exposed to normal blood circulation and their abdominal cavity was carefully placed on aluminium sheets. We immediately poured isopentane-propane cryogen (− 193 °C) precooled in liquid nitrogen over the small intestines from the outer side of the serous membrane^52,53^. The frozen intestinal tissues were commonly processed for freeze substitution, and finally embedded in paraffin wax, as previously reported^54^. All paraffin-embedded tissues were cut transversely to 5 μm-thick sections. The thin sections were routinely stained with haematoxylin and eosin for tissue morphological analysis under well-freezing condition using a light microscope. The sections were then processed for immunohistochemical analysis by incubation in 1% hydrogen peroxide in PBS and 2% gelatine (Sigma, St Louis, MO, USA) in PBS for 1 h. Then, the sections were immunostained with several different primary antibodies in PBS at room temperature for 2 h. Primary antibodies (Table 2) were rabbit or goat polyclonal antibodies against mouse PGP9.5, synaptophysin, and c-Kit. Immunocontrols were prepared by incubating the thin sections in 2% gelatine without the primary antibodies. They were then incubated with horseradish peroxidase-conjugated donkey anti-rabbit or anti-goat IgG (H + L) antibody at a dilution of 1:400 (Abcam, Cambridge, UK) at room temperature for 1 h, followed by visualisation with cobalt-enhanced diaminobenzidine (DAB) in buffer solution containing hydrogen peroxide (Thermo Fisher Scientific Pierce, Rockford, IL, USA). The immunostained sections were additionally incubated in 0.04% osmium tetroxide in 0.1 M phosphate buffer for 30 s to enhance the contrast of the DAB reaction products, as previously reported^55^. For immunofluorescence, some of the rehydrated thin sections were first incubated with 2% gelatine in PBS, followed by incubation with synaptophysin and c-Kit at dilutions of 1:50 and 1:400 for 2 h, respectively. The, the sections were incubated with Alexa Fluor 488-conjugated donkey anti-rabbit IgG antibody and Alexa Fluor 594-conjugated donkey anti-goat IgG antibody (Invitrogen, Eugene, OR, USA) at a dilution of 1:400 for 1 h each, and finally washed in PBS. To detect nuclei, the specimens were incubated in VECTASHIELD mounting medium with DAPI (Vector Laboratories, UK). Finally, the sections were observed under a BZ-X700 fluorescence microscope (Keyence, Osaka, Japan).

**Table 2:** List of primary antibodies used in this study.

Target molecule	Host	Manufacturer	Dilution
PGP9.5	Rabbit	Proteintech, IL, USA	1:400
Synaptophysin (SP11)	Rabbit	Thermo Fisher Scientific, Rockford, IL, USA	1:50
c-kit	Goat	R&D Systems Inc., MN, USA	1:400

### Whole-mount preparations

To produce whole-mount preparations, samples were fixed in 4% paraformaldehyde in 0.1 M phosphate buffer overnight at 4 °C. The specimens were first rinsed in DMSO for 30 min and then in PBS. After removing the circular muscle layers, isolated longitudinal muscle layers attached to the myenteric plexus were placed in PBS containing 0.3% Triton-X for 20 min^56,57^, followed by pre-incubation with 2% gelatine in PBS for 1 h. Then, the specimens were incubated with primary antibodies (synaptophysin, 1:50 and c-Kit, 1:400) at 4 °C overnight, followed by rinsing and incubation with secondary antibodies conjugated to Alexa 488 and Alexa 594 at a dilution of 1:400 for 1 h. To detect nuclei, the specimens were incubated in VECTASHIELD mounting medium with DAPI. The stained specimens were observed under the BZ-X700 fluorescence microscope, and whole-mount images were taken using the 40 × objective lens.

### Statistical analysis

Statistical analyses were performed using GraphPad Prism version 6.07 for windows software (GraphPad Software Inc., La Jolla, CA, https://www.graphpad.com/). Unpaired Student’s *t*-test was used to compare weights (Fig. 1b), weight gains (Fig. 1c), and blood glucose levels (Fig. 1d). Serial section images were reconstructed into 3D images and axons were randomly selected in the myenteric plexus. Varicosities (Fig. 4j,k), mitochondria (Fig. 4l–n), and lysosomes (Fig. 4o–q) were measured using the area list and the polyline tools in TrakEM2 software. One-way ANOVA followed by Bonferroni (Fig. 4j,l–n) or Kruskal–Wallis (Fig. 4k,o–q) post-hoc tests were utilised for multiple comparisons. PGP9.5 foci in the myenteric plexus per transverse section were counted in a blinded fashion (Fig. 5s). Data from all examined sections were used to calculate the percentage area of positive pixels relative to the total examined area in pixels (Fig. 5t). For semiquantitative analysis, synaptophysin scores (Fig. 5u) were calculated (0, no staining; 1, weak; 2, mild; 3, strong) by considering the immunostaining intensity in the tissue environment, as we have previously reported^58^. The synaptophysin density index (Fig. 6j) illustrates the standard thresholding procedure on a boxel image (10 × 10 μm) of synaptophysin-labelled nerve fibres from the myenteric plexus, without nucleus. A thresholding, using the standard threshold function within Fiji, was obtained. Synaptophysin density index calculations in a boxel image (stained area/myenteric plexus area) were performed. To compare means of STD-Veh, HFD-Veh, and HFD-PLZ groups, multiple comparisons by one-way ANOVA followed by Bonferroni or Kruskal–Wallis post-hoc tests were used (Figs. 4, 5, 6). Significant differences were determined as *P* < 0.05, and values were expressed as the mean (standard deviation) or median (interquartile range) for normally and non-normally distributed data, respectively.

## Supplementary information

Supplementary information.
