# Supplementary material for: Sodium-glucose co-transporter (SGLT) inhibitor restores lost axonal varicosities of the myenteric plexus in a mouse model of high-fat diet-induced obesity
Source: Sci Rep. 2020 Jul 23;10:12372. doi: 10.1038/s41598-020-69256-9 (PMC7378553; doi:10.1038/s41598-020-69256-9)
Supplement: Supplementary file 1 — Supplementary information. [file 41598_2020_69256_MOESM1_ESM.docx]

**Sodium-glucose co-transporter (SGLT) inhibitor restores lost axonal varicosities of the myenteric plexus in a mouse model of high-fat diet-induced obesity**

Satoshi Shimo^1,^*, Sei Saitoh^2^, Huy Bang Nguyen^3,4,5^, Truc Quynh Thai^3,4,6^,

Masako Ikutomo^7^, Ken Muramatsu^8^, & Nobuhiko Ohno^3,9^

^1^Department of Occupational Therapy, Health Science University, 7187 Kodachi, Fujikawaguchiko, Yamanashi 401-0380, Japan

^2^Department of Anatomy II and Cell Biology, Fujita Health University School of Medicine, 1-98 Dengakugakubo, Kutsukake-cho, Toyoake, Aichi 470-1192, Japan

^3^Division of Neurobiology and Bioinformatics, National Institute for Physiological Sciences, 38 Saigonaka, Meidaiji-cho, Okazaki, Aichi 444-8787, Japan

^4^Department of Anatomy and Structural Biology, Graduate School of Medical Science, University of Yamanashi, 1110 Shimokato, Chuo, Yamanashi 409-3898, Japan

^5^Department of Anatomy, Faculty of Medicine, University of Medicine and Pharmacy (UMP), 217 Hong Bang, District 5, Ho Chi Minh 70000, Vietnam

^6^Department of Histology Embryology Genetics, Faculty of Basic Medical Sciences, Pham Ngoc Thach University of Medicine, 02 Duong Quang Trung, District 10, Ho Chi Minh 70000, Vietnam

^7^Department of Physical Therapy, University of Tokyo Health Sciences, 4-11 Ochiai, Tama, Tokyo 206-0033, Japan

^8^Department of Physical Therapy, Kyorin University Faculty of Health Sciences, 5-4-1 Shimorenjaku, Mitaka, Tokyo 181-8612, Japan

^9^Department of Anatomy, Division of Histology and Cell Biology, Jichi Medical University, School of Medicine, 3311-1 Yakushiji, Shimotsuke, Tochigi 329-0498, Japan

*Corresponding author:

Satoshi Shimo

Department of Occupational Therapy, Health Science University: 7187 Kodachi, Fujikawaguchiko, Yamanashi 401-0380, Japan

Tel: +81-555-83-5200; Fax: +81-555-83-5100; E-mail: sshimo@kenkoudai.ac.jp


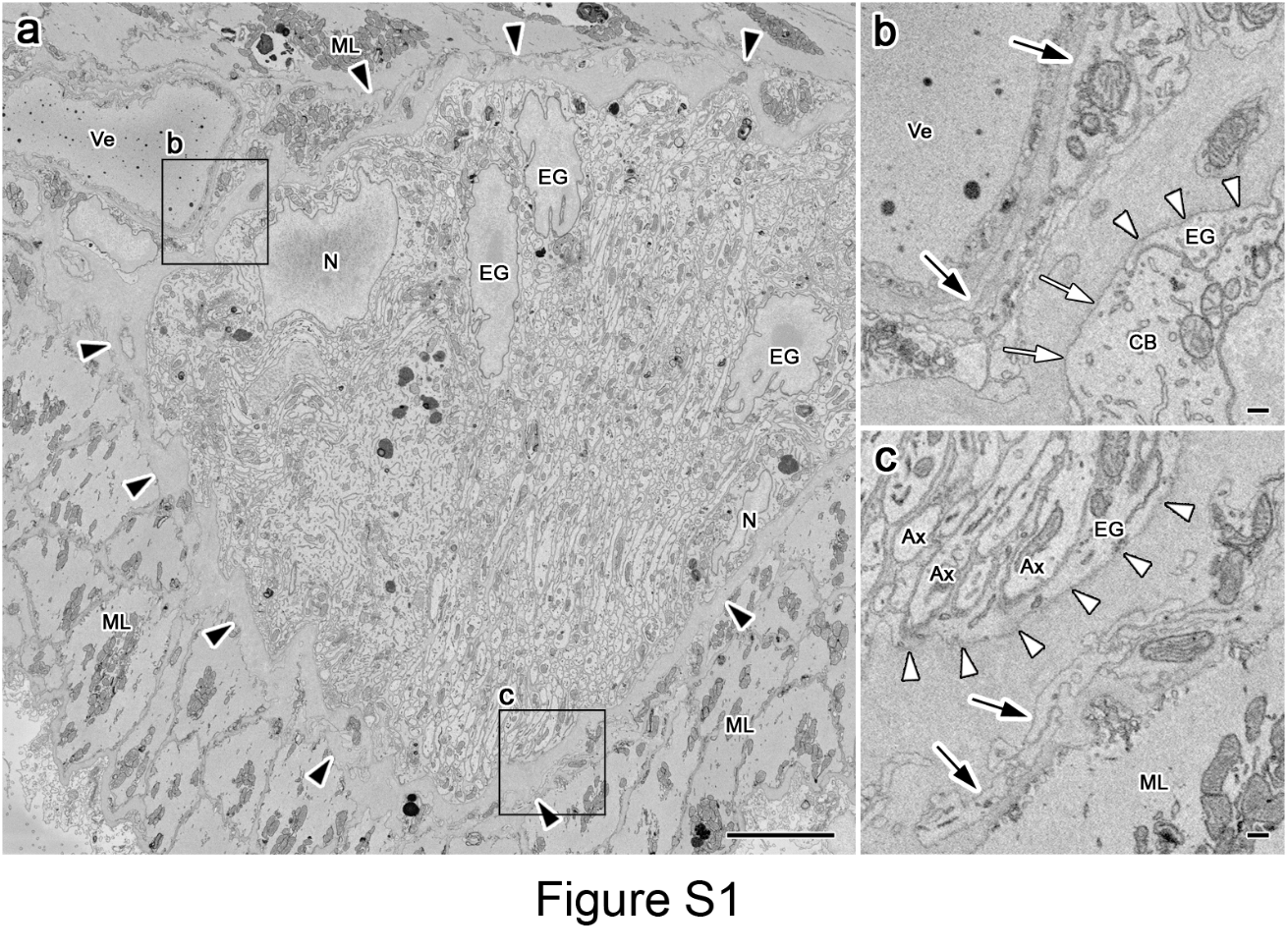


**Figure S1.** Analysis of the myenteric plexus in STD-Veh mice. **a** The myenteric plexus is located between the longitudinal and circular smooth muscle layers. The ganglia were completely surrounded by a basal lamina and isolated from the connective tissues (arrowheads) and blood vessels (Ve). **b-c** Processes of fibroblast-like cells (arrows) are present in the extraganglionic space. **b** Parts of the nerve cell body (CB) reach the surface of the ganglion (white arrows), where they are exposed to the extraganglionic space without a covering of glial cytoplasm (white arrowheads). **c** Within the plexus, enteric glia (EG) are molded over the surface of the adjacent neural structures. Ax: axon, EG: enteric glial cell, N: neuron, ML: muscular layer. Bars: (**a**) 5 µm, (**b**, **c**) 500 nm.

**
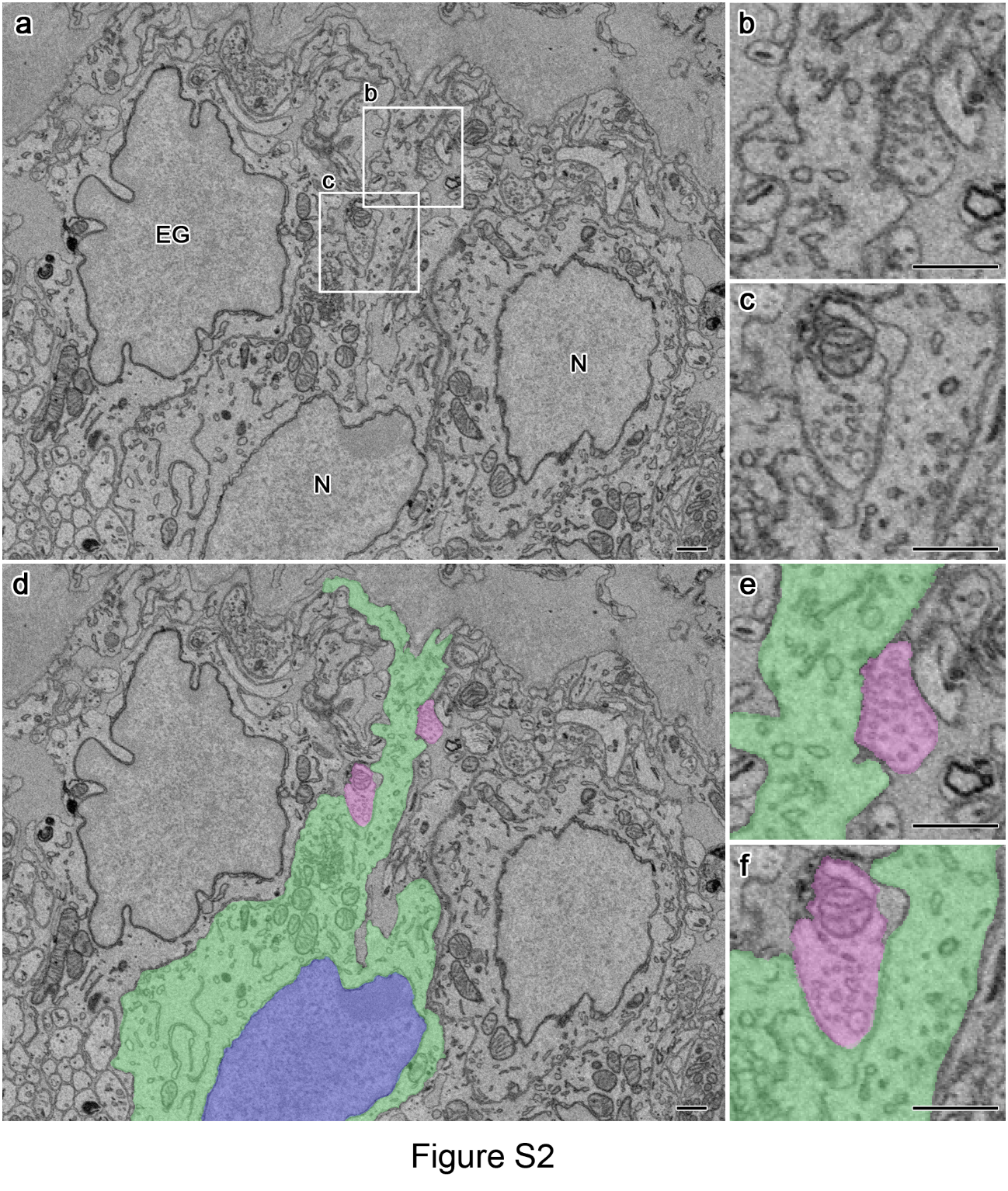
**

**Figure S2**. The axons within the ganglia around granular vesicles were presumably the processes of an intramural neuron in STD-Veh mice. **a** Electron microscopy characterizes enteric glial cell (EG), neurons (N), and axon ultrastructure within in the small intestine of STD-Veh mice. **b-c** The myenteric plexus is highly magnified, corresponding to the rectangular areas in (**a**). **d-f** axon (red), processes of an intramural neuron (green), and nucleus of neuron (blue) were marked in segmentation, and traced using TrakEM2. Bars: 500 nm.
